# Pupil Size Associated with the Largest Iris Volume in Normal Chinese Eyes

**DOI:** 10.1155/2018/8058951

**Published:** 2018-12-27

**Authors:** Shuning Li, Jing Jiang, Yiquan Yang, Gewei Wu, Shi-Ming Li, Ravi Thomas, Ningli Wang

**Affiliations:** ^1^Beijing Tongren Eye Center, Beijing Tongren Hospital, Beijing Ophthalmology & Visual Science Key Lab, Capital Medical University, Beijing, China; ^2^Fushun Eye Hospital, Fushun, China; ^3^Department of Ophthalmology, Beijing Shijingshan Hospital, Beijing, China; ^4^Queensland Eye Institute, Brisbane, Australia; ^5^University of Queensland, Brisbane, Australia; ^6^Beijing Tongren Eye Center, Beijing Tongren Hospital, Beijing Ophthalmology & Visual Science Key Lab, Beijing Institute of Ophthalmology, Capital Medical University, Beijing, China

## Abstract

**Background:**

To determine the range of pupil size that has the largest iris volume in normal eyes.

**Methods:**

31 healthy adult Chinese volunteers underwent swept-source anterior segment OCT examination in both eyes. Pilocarpine 1% was instilled in a randomly selected eye (eye with induced miosis (ME)) of each participant to obtain iris volume (IV) measurements over a range of pupil sizes. OCT was performed prior to and one hour after pilocarpine in both ME and fellow eye (FE). Iris volume (IV), anterior chamber volume (ACV), anterior chamber depth (ACD), and pupil size (PS) were recorded. A scatter plot was used to depict the association between each pupil size and IV.

**Results:**

The pupillary sizes for which IV was recorded in ME and FE ranged from 1.161 mm to 6.665 mm. The mean IV increased with miosis in both ME and FE; in 13 eyes, IV decreased with a decrease in pupillary size. PS between 3.812 and 6.665 mm was associated with an increase in IV, while PS between 3.159 and 5.54 mm was associated with a decrease. The relationship between PS and IV was in the shape of a downward parabola and was modeled using a quadratic equation (*y* = −1.3121*x*^2^ + 8.8429*x* + 16.423, *R*^2^ = 0.26886). The largest IV occurred at PS between 3 and 4 mm.

**Conclusions:**

The relationship between PS and IV in this study was in the shape of a downward parabola. The largest IV was recorded at a pupillary size between 3 and 4 mm. This trial is registered with ChiCTR-ROC-17013572.

## 1. Introduction

Primary angle-closure glaucoma (PACG) is one of the major causes of glaucoma and blindness in China [1]. It is estimated that there will be more than 10 million affected by PACG in China by 2020 accounting for 48% of the total number of PACG cases worldwide [2].

Primary angle-closure suspects (PACSs) are those with angles at risk for but with no evidence of primary angle-closure disease (PACD). PACD includes primary angle closure (PAC), primary angle-closure glaucoma (PACG), and acute attack (AAC) [3, 4].

The cause of PACD is multifactorial [5, 6]. Some of the known risk factors include short axial length, a shallow anterior chamber, a thicker lens, and a more anteriorly positioned lens [7]. Ultrasound biomicroscopy (UBM) and anterior segment OCT (AS-OCT) have identified ocular biometric parameters such as anterior chamber width, anterior chamber area and volume, iris thickness, iris area, lens curvature, and lens vault as risk factors [8–19]. More recently, the importance of dynamic changes in the iris in PACD has also been recognized [20–29].

Not all PACSs or PACDs progress. A population-based study from South India reported that, over a 5-year period, 22% (95% CI 9.8–34.2%) of PACSs progressed to PAC, while 28.5% of PAC (95% CI 12–45%) progressed to PACG [30, 31]. The Zhongshan Angle Closure Prevention Trial from China has studied the role of laser iridotomy in PACS, but the results have not yet been published [32]. In a clinical setting, however, most PACSs receive an LPI. In order to avoid overtreatment and decrease unnecessary costs, it is important to study the mechanisms and better identify those who are at higher risk of progression.

Iris volume (IV) and its change with pupil size (PS) have been reported as an important risk factor for PACD [21, 24, 25, 28, 33]. The formula for measurement of IV using AS-OCT had significant shortcomings and led to the use of iris area instead [27]. The measurement of IV with swept-source OCT is however more accurate [24]. The iris area and IV have been studied over a range of pupil size (PS) by inducing mydriasis [21, 24, 25, 27, 28, 33–35]. Mydriasis and light-induced miosis alone could not provide a large spectrum of PS over which changes in IV can be measured and its relationship with PS can be determined. Observations of IV over smaller PS induced by pharmacological miosis, while not physiological, may provide useful information about the relationship of iris volume with pupillary size and the PS at which IV is the maximum in normal people. The purpose of this study is to report the change in IV from physiological PS to pharmacologically induced miotic PS in healthy volunteers and assess the PS at which IV is the maximum.

## 2. Materials and Methods

Thirty-one healthy volunteers from the staff of the Beijing Tongren Hospital were recruited for the study. All participants underwent a comprehensive ocular examination that included visual acuity test, refraction test, intraocular pressure (IOP) measurement using Goldmann applanation tonometry, slit-lamp microscopy, fundus examination, fundus photography (CR-DGi with a 20D SLR back; Canon, Tokyo, Japan), and optical coherence tomography (OCT; Optovue, CA, USA). All had open angles as determined by swept-source OCT of the anterior segment (CASIA SS-1000 OCT®; Tomey Inc., Nagoya, Japan). Those with any ocular disease, refractive error > +6.00 diopters or −6.00 diopters, pigment dispersion syndrome, and pseudoexfoliation were excluded. The study was approved by the Ethics Committee of the Beijing Tongren Hospital and conducted in accordance with the Declaration of Helsinki. All participants provided an informed consent.

Both eyes of all participants underwent swept-source OCT examination of the anterior segment (CASIA SS-1000 OCT®; Tomey Inc., Nagoya, Japan). A single operator performed all scans as described in the manual and the literature; the examination was performed in ambient illumination [24]. In brief, the participants placed their chin on the chin rest and were asked to fixate on the internal fixation target while scanning was performed. In order to avoid eyelid artifacts, the operator exerted mild traction on the eyelids where needed.

A drop of pilocarpine 1% (Xingqing, China) was then instilled in one eye of each participant chosen randomly by the toss of a coin; an hour later, the pupil size was measured and the scan was repeated in both eyes. The eye for which miosis was induced is referred to as ME, and the fellow eye is referred to as FE. All images with lid or motion artifacts were excluded. The built-in software automatically located the scleral spur and calculated pupil size (PS), anterior chamber depth (ACD), anterior chamber volume (ACV), and iris volume (IV).

In order to obtain a wider range of pupillary diameters, pre- and postmiosis PS in both ME and FE was used for analysis, but FE pupils that were more dilated on the second examination were excluded. The relationship between IOP and pupil size before instillation of pilocarpine was depicted in a scatter plot and modeled using linear regression analysis. The relationship between IV and pupil size was depicted in a scatter plot and modeled using both linear regression analysis and a quadratic equation. Pre and postmiosis parameters in both ME and FE were compared using the paired *t*-test, and *P* < 0.05 was considered statistically significant. SPSS statistical software version 22.0 (SPSS, Inc., Chicago, IL) was used for data analysis.

## 3. Results

Table 1 shows the demographic and baseline characteristics of the 31 subjects (62 eyes) enrolled in the study. There were 5 (16%) males and 26 (84%) females with a mean age of 35.48 ± 14.46 years. Baseline results showed no statistically significant differences in IOP, PS, ACV, IV, and ACD between ME and FE. 15 right and 16 left eyes were randomly selected by the toss of a coin for induction of miosis.

**Figure 1:**
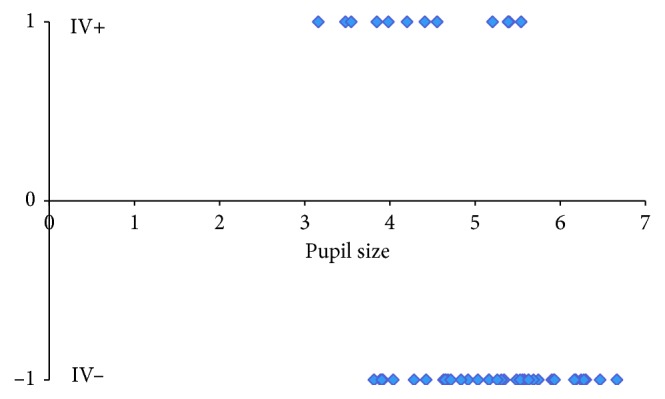
Baseline pupil size by an increase or decrease in IV at one hour after induction of miosis (IV− represents decreasing IV and IV+ represents increasing IV, *n*=52).

One hour following instillation of pilocarpine, the pupil size decreased in all ME eyes. There was a trend to smaller pupil sizes in FEs also, but in 10 eyes, the pupil size increased from that at baseline. As the objective was to determine IV in eyes with decreased pupil size, these 10 eyes with an increase in PS were excluded. The IV decreased with smaller pupil size in 9 of 31 MEs and 4 of 21 FEs (Table 2). The range of baseline pupil size for eyes whose IV increased with miosis was 3.812–6.665 mm; it was 3.159–5.54 mm for those whose IV decreased. At pupil sizes between 3.889 and 5.54 mm, the IV increased in 16 eyes and decreased in 7. To better describe the individual responses of IV to the decrease in pupillary size, the eyes were divided into two groups: IV+ for those with an increase in IV following miosis and IV− for those in whom it decreased. The relationship of the baseline pupil size and the increase or decrease in IV is shown in Figure 1.

**Figure 2:**
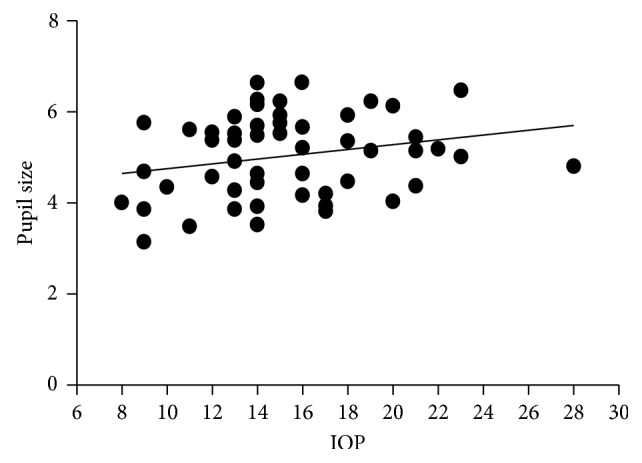
Scatter plot between IOP and pupil size before instillation of pilocarpine (*n*=62). Linear regression model: *y* = 0.05366 ∗ *x* + 4.221 (*P*=0.543).

The relationship between IOP and pupil size before instillation of pilocarpine is shown in Figure 2; the linear regression model was *y* = 0.05366 ∗ *x* + 4.221 (*P*=0.543).

**Figure 3:**
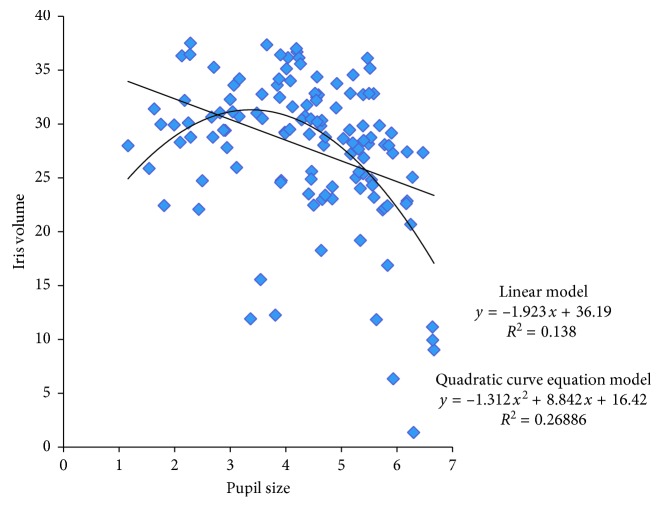
Scatter plot of pupil size and IV for ME and FE before and after miosis (*n*=124). ME: eyes with induced miosis; FE: fellow eyes.

A scatter plot showing the relationship between pupil size and IV at baseline and one hour later in all eyes of both ME and FE is shown in Figure 3. With pupil sizes between 1.161 mm and 6.665 mm, the linear regression model was *y* = −1.923*x* + 36.19 (*R*^2^ = 0.138). The relationship was then modeled using a quadratic equation *y* = −1.3121*x*^2^ + 8.8429*x* + 16.423 (*R*^2^ = 0.26886). The higher coefficient of determination with the quadratic equation suggests that this is the better fit. The quadratic model suggests that the relationship of IV with pupil size is a downward parabola with the largest IV at pupil sizes between 3 and 4 mm.

The difference in parameters at baseline and one hour following instillation of pilocarpine for ME and FE is shown in Table 3. In the ME, IOP decreased from 15.21 ± 3.43 mmHg to 13.81 ± 3.31 mmHg, PS decreased from 5.02 ± 0.92 mm to 3.08 ± 1.19 mm, ACV decreased from 152.69 ± 39.41 mm^3^ to 140.48 ± 35.24 mm^3^, ACD decreased from 2.86 ± 0.35 mm to 2.79 ± 0.34 mm, and IV increased from 26.21 ± 7.63 mm^3^ to 30.02 ± 4.40 mm^3^. In the FE, IOP also decreased from 15.00 ± 4.35 mmHg to 13.71 ± 3.60 mmHg, PS decreased from 5.05 ± 0.87 mm to 4.37 ± 1.15 mm, ACV decreased from 149.63 ± 38.66 mm^3^ to 144.66 ± 37.32 mm^3^, ACD decreased from 2.84 ± 0.36 mm to 2.81 ± 0.39 mm, and IV increased from 26.79 ± 7.38 mm^3^ to 28.05 ± 1.21 mm^3^.

## 4. Discussion

In predisposed eyes, dilatation of the pupil is a risk factor for angle closure (AC), especially acute angle closure [36]. Pupillary block is maximized in the mid-dilated position of the pupil [37]. A formula to calculate pupillary block force (PBF) showed that the change in PBF with the change in pupil size from 2 to 6 mm was in the shape of a downward parabola with the largest PBF occurring with pupil sizes of 3 to 5 mm [36]. The resultant bowing of the iris with narrowing of the angle has been demonstrated in recent studies using newer imaging technologies such as UBM and AS-OCT [38–40].

Iris area and volume contribute to the shape of the iris [34]. The known decrease of iris area with pupillary dilatation is related to the loss of fluid from the iris stroma, and this decrease is less in eyes with angle closure as compared to normal eyes [34]. IV results from the combination of iris length and thickness as well as the ability of fluid to pass through the stroma [34]. The change in IV with range of PS that results in the highest IV is important in order to further understand primary angle closure.

In order to exclude the effect of IOP on PS, the relationship between IOP and PS was analyzed. The linear regression model, *y* = 0.05366 ∗ *x* + 4.221 (*P*=0.543), suggests that IOP did not affect PS in this sample and does not need to be considered in our investigation of the relationship between IV and PS.

With the use of pilocarpine, we were able to obtain pupil sizes from 1.161 mm to 6.665 mm, a range that was wide enough to investigate the relationship of iris changes with variation in PS. Following miosis, the PS decreased in all ME eyes and in 21 of 31 (68%) FEs. A significant increase in IV occurred in ME but not in FE. It has been shown that iris area and volume decrease on dilatation [24, 27, 28, 34, 35]. We would expect the opposite result with miosis, and that is what our results generally show.

While the mean IV increased with miosis, such an increase was not seen in all miotic pupils: with smaller PS, the IV decreased in 9 of 31 MEs and 4 of 21 FEs. It is clear that IV does not necessarily increase with a decrease in pupil size. Iris volume decreases with pupillary dilatation, but this is also not true for all eyes [24, 33]. The IV decreased in 70 of 86 eyes (81.4%) when measured in light versus dark conditions but increased in 16 eyes (18.6%) [24]. An increase in iris volume was reported in 19 of 21 (90%) fellow eyes of patients with acute angle closure when the pupils changed from the light to dark conditions and after pharmacologic dilation in 28 of 30 eyes (93.3%) [33]. The formula used to calculate IV in the above study has been shown to have shortcomings [27]. It seems that, while there are trends toward a decrease in IV with dilatation, in individual eyes, it may increase or decrease with miosis or mydriasis. As shown in Figure 1, the baseline PS of those with an increase in IV ranged from 3.812 to 6.665 mm, while it decreased in those with a PS from 3.159 to 5.54. With baseline pupil sizes between 3.812 and 5.54 mm, the IV either increased (16 eyes) or decreased (7 eyes).

An important finding in this study is that the relationship between PS and IV is in the shape of a downward parabola. A linear regression model was used initially followed by a quadratic model. The difference in fit may be open to question, but the coefficient of determination is better with the quadratic equation. The range of PS (1.161–6.665) at which IV was measured covers the mid-dilation spectrum (2 to 6 mm) at which pupillary block force is assumed to be maximum [37]. The curve of the downward parabola that represents IV is maximum at PS of 3 to 4 mm; that is, as shown in Figure 2, the largest IV occurs in this range. The figure also suggests an explanation for why IV can increase or decrease. If the largest IV is present with a PS of 4 mm, then either mydriasis or miosis will result in decreasing IV. If the IV is on the right side of the downward parabola, miosis may produce an increase in IV and mydriasis may produce a decrease. If IV is on the left side of the parabola, miosis may decrease IV while mydriasis may increase it. These changes in IV are consistent with the fact that AC is most likely in the mid-dilated position of the pupil—the largest IV that occurs at this PS and contributes toward narrowing of the angle and predisposes to AC.

Our results are different from those of studies that have reported a linear relationship between IV and PS [28, 33]. One possible reason is the inaccuracy of the formula that was used to calculate IV [27]. Another reason could be that the ranges of pupil sizes 2.98 ± 0.67 mm to 6.32 ± 0.94 mm in Aptel's study and 3.99 ± 0.65 mm to 7.26 ± 0.53 mm in Zhang's study were not wide enough to demonstrate the phenomenon we have described [28, 33]. A linear relationship of IV with PS may be true within a certain range of PS, but such a relationship over the whole range of PS is difficult to reconcile with the fact that AC occurs most frequently with mid-dilated pupils, while the largest IV occurs with the smallest PS.

The study has several limitations. Only normal people were recruited for this preliminary study. The behavior of IV in PACS and PACD is likely to be different and requires further study. It would also be ideal, but more difficult, to record the changes in IV with changes in PS in the same eye. Moreover, using pilocarpine to induce miosis is not physiological, and its extrapolation to AC may not be entirely accurate. The study was however intended to determine the range of pupil size with the largest IV; pharmacologically induced miosis provided the range of pupil sizes required to study this. Finally, although the pattern of the relationship of IV with PS is discernable, the sample size is small.

In conclusion, the relationship between iris volume and pupillary sizes between 6.65 and 1.161 mm in normal adult Chinese eyes with pharmacologically induced miosis is in the shape of a downward parabola, with the largest iris volume occurring with a PS of 3 to 4 mm. This means IV increases from baseline levels with pilocarpine-induced miosis but is not consistent for all eyes which can be explained by the shape of the relationship described.

## Figures and Tables

**Table 1:** Demographic and baseline characteristics (*n*=31).

Characteristics	Miosis-induced eye (ME)	Fellow eye (FE)	*P*
Age (years)	35.48 ± 14.46		—
Gender (female), *n* (%)	26 (84%)		—
Eye (right), *n* (%)	15 (48%)		—
IOP (mmHg)	15.21 ± 3.43	15 ± 4.35	0.7
Pupil size (mm)	5.02 ± 0.92	5.05 ± 0.87	0.913
Anterior chamber volume (mm^3^)	152.69 ± 39.41	149.63 ± 38.66	0.737
Iris volume (mm^3^)	26.21 ± 7.63	26.79 ± 6.38	0.766
Anterior chamber depth (mm)	2.86 ± 0.35	2.84 ± 0.36	0.759

**Table 2:** Range of pupil size at baseline by an increase or decrease in IV one hour after induction of miosis (*n*=52).

	ME	FE	Pupil size range (mm)
Increase in IV	22 (71%)	17 (81%)	3.812–6.665
Decrease in IV	9 (29%)	4 (19%)	3.159–5.54

ME: miosis-induced eye; FE: fellow eye; IV: iris volume. 10 FE eyes were excluded as explained in the text.

**Table 3:** Parameters at baseline and at one hour after induction of miosis (*n*=31).

Variables	ME			FE		
	Baseline	After one hour	*P*	Baseline	After one hour	*P*
IOP (mmHg)	15.21 ± 3.43	13.81 ± 3.31	**0.003**	15.00 ± 4.35	13.71 ± 3.60	**0.018**
PS (mm)	5.02 ± 0.92	3.08 ± 1.19	**0.001**	5.03 ± 0.87	4.37 ± 1.15	**0.001**
ACV (mm^3^)	152.69 ± 39.41	140.48 ± 35.24	**0.001**	149.63 ± 38.66	144.66 ± 37.32	**0.023**
IV (mm^3^)	26.21 ± 7.63	30.02 ± 4.40	**0.002**	26.79 ± 7.38	28.05 ± 1.21	0.112
ACD (mm)	2.86 ± 0.35	2.79 ± 0.34	**0.001**	2.84 ± 0.36	2.81 ± 0.39	0.134

ME: miosis-induced eye; FE: fellow eye; IV: iris volume.

## Data Availability

The data used to support the findings of this study are available from the corresponding author upon request.
